# Increasing Role of FDG-PET/CT in Detecting Bone Marrow Metastasis of Solid Tumors in Adults: An Analysis of Ten Patients

**DOI:** 10.4021/wjon598w

**Published:** 2013-01-04

**Authors:** Fatih Selcukbiricik, Ozcan Yildiz, Sabire Yilmaz, Deniz Tural, Hande Turna, Nil Molinas Mandel, Nukhet Tuzuner, Suheyla Serdengecti, Metin Halac

**Affiliations:** a Cerrahpasa Medical Faculty, Istanbul University, Kocamustafapasa, Istanbul, Turkey; b Department of Medical Oncology, Istanbul University, Kocamustafapasa, Istanbul, Turkey; c Department of Nuclear Medicine, Istanbul University, Kocamustafapasa, Istanbul, Turkey; d Department of Pathology, Istanbul University, Kocamustafapasa, Istanbul, Turkey

**Keywords:** Bone metastasis, Bone marrow metastasis, FDG-PET/CT, Solid tumors

## Abstract

**Background:**

We aimed to test the hypothesis that whether FDG-PET/CT which was ordered for various purposes can predict suspected or particularly unsuspected bone marrow metastasis (BMM) from the complete blood count and therefore can change the management of these patients.

**Methods:**

In this retrospective study, the study sample consisted of 68 subsequent patients presented to our institution’s pathology department with bone marrow metastases of solid tumors. PET/CT was found to have been ordered in 10 out of 68 patients (6.8%) for various purposes. All patients gave informed consent about the PET/CT examinations and bone marrow biopsies.

**Result:**

FDG-PET/CT was ordered in 10 out of 68 solid tumor patients with pathologically proven BMM. Of these 10 patients, 3 were female and 7 were male; mean age was 54.7 years. While FDG PET/CT showed bone and BMM in 4 of 10 patients (40%), the rest of the patients had BMM without bone involvement. Five patients (50%) who had probable bone marrow involvement on their FDG PET/CT scans had unsuspected complete blood counts with regard to BMM.

**Conclusion:**

PET/CT has the ability to detect a substantial number of metabolically active tumor cells in the bone marrow in all of our patients which we proved by bone marrow biopsies. We think that this cohort of patients with solid tumors is hypothesis-generating with regard to detecting early bone marrow metastases by FDG PET/CT.

## Introduction

Bone marrow metastasis (BMM) of solid tumors is a rather rare but a relatively early phenomenon. Stem cells expressing CD44, a hyaluronic acid cell surface receptor, has been accused for the early deposition of malignant cells in the bone marrow [1] BMM poses a clinical challenge to the oncologist in tailoring chemotherapy options and dosing especially when complete blood counts are compromised. Therefore, early diagnosis of BMM of solid tumors is of utmost importance depending on the primary solid tumor. For decades, conventional methods such as bone scintigraphy, MR, CT and also FDG PET and FDG PET/CT have been used to show bone and bone marrow metastasis. These imaging modalities can be divided into two groups based on the differences in physical and biological principles as those which detect disease site at bone marrow and osteoblastic reaction after invasion of surrounding bone by the pathologic process. The former one was further subclassified into two groups as those that visualize lesions as negative focal marrow defects seen on BMS or MR and those that directly visualize abnormal tissue such as with FDG PET/CT [1]. In addition to imaging modalities other methods such as low mean platelet volume (MPV) and meticulously prepared touch imprint smears [2] or circulating tumor cells (CTCs) [3] have been studied in demonstrating BMM. FDG PET/CT has the advantage of giving information about primary tumor site, lymph node status, and other organ metastasis in a single study, which has been regarded as a “quantum jump” in nuclear medicine. In addition, it has directed attention to the bone marrow not only to search for unsuspected deposits but also to perform high yield bone marrow biopsies at least in lymphomas and multiple myeloma [4, 5].

Skeletal metastasis usually happens by seeding of tumor emboli through bloodstream and also via retrograde venous blood flow or direct extension [6]. Bone remodeling due to osteolytic and osteoblastic reactions form the basis of indirect evidence of skeletal metastasis in solid tumors. Whereas, the superiority of the FDG PET/CT in detecting bone marrow metastasis comes from the fact that it visualizes the metabolic activity of disseminated tumor cells directly in the bone marrow unlike bone marrow scintigraphy or other radiological methods which provides indirect evidence of skeletal metastasis [1].

### Purpose

We aimed to test the hypothesis that whether FDG-PET/CT which was ordered for various purposes can predict suspected or particularly unsuspected BMM from the complete blood count and therefore can change the management of these patients.

## Patients and Methods

In this retrospective cohort study, the study sample consisted of 68 subsequent patients presented to our institution’s pathology department with bone marrow metastases of solid tumors from January 2006 to March 2010. We searched the patient files to detect which patients had undergone FDG-PET/CT analysis. PET/CT was found to have been ordered in 10 out of 68 patients (6.8%) for various purposes. These patients were included in the final analysis. The interval between the PET/CT examination and bone marrow biopsy was approximately 2 weeks. Seven of 10 patients were chemotherapy naive at the time of analysis whereas 3 patients had received chemotherapy. All patients gave informed consent about the PET/CT examinations and bone marrow biopsies. The detailed excerpts of the patients are as follows.

### Patient 1

**Figure 1:**
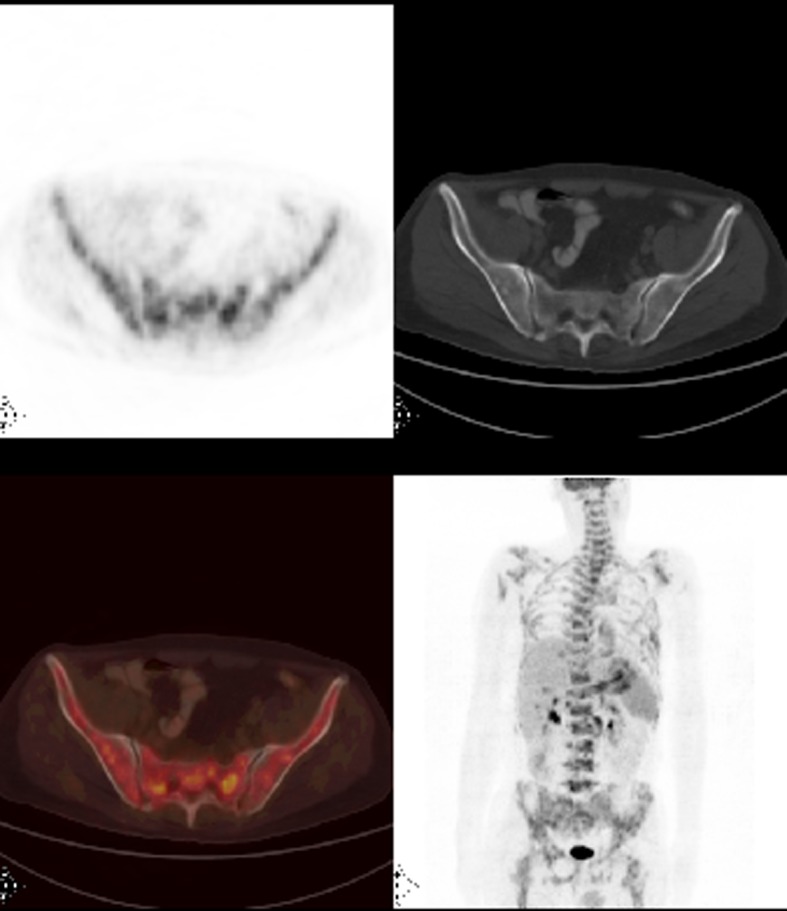
Axial PET/CT and MIP (maximum intensity projection) FDG PET/CT images revealed heterogeneously widespread increased FDG uptake at the skeleton which suggests bone marrow involvement.

A 46-year-old male patient presented with fatigue, weight loss, anemia and dyspeptic complaints for the last 2 months. Gastric cancer was diagnosed with upper endoscopic biopsy. FDG-PET/CT was ordered in order to initial staging. PET/CT images not only demonstrated increased FDG accumulation at the primary tumor site but also revealed multiple metastatic intraabdominal lymphadenopathies and left supraclavicular lymph node. Heterogeneous widespread FDG uptake in the axial skeleton suggested bone marrow involvement (Fig. 1). Hgb was 9.5 g/dL; WBC, 4,100 and PLT, 33,000 per µL. Bone marrow biopsy showed metastatic gastric cancer.

### Patient 2

A 24-year-old female patient was diagnosed with embryonal rhabdomyosarcoma of the perineum. FDG PET/CT which was ordered for restaging showed hypermetabolic mass at the perineal region consistent with recurrence and bilateral inguinal metastatic lymph nodes. Additionally, there was left iliac lytic lesion with increased FDG uptake consistent with bone-bone marrow metastases and moderately heterogenous disseminated intense FDG accumulation in the axial skeleton suggestive of bone marrow involvement. Supported by prolonged cytopenia PET/CT findings prompted us to do a bone marrow biopsy which proved to have metastatic rhabdomyosarcoma.

### Patient 3

A 59-year-old female patient having a history of right modified radical mastectomy 16 years previously due to breast cancer had developed bone metastases and 6 cycles of chemotherapy were administered. After the completion of chemotherapy, FDG-PET/CT was ordered to evaluate prolonged thrombocytopenia. PET/CT showed moderately heterogenous increased FDG uptake in the axial skeleton suspicious of bone marrow involvement. Bone marrow biopsy proved solid metastasis with carcinoma consistent with breast cancer. Her Hgb was 9.6 g/dL, WBC 7,400 and platelets 78,000 per µL at the time of biopsy.

### Patient 4

**Figure 2:**
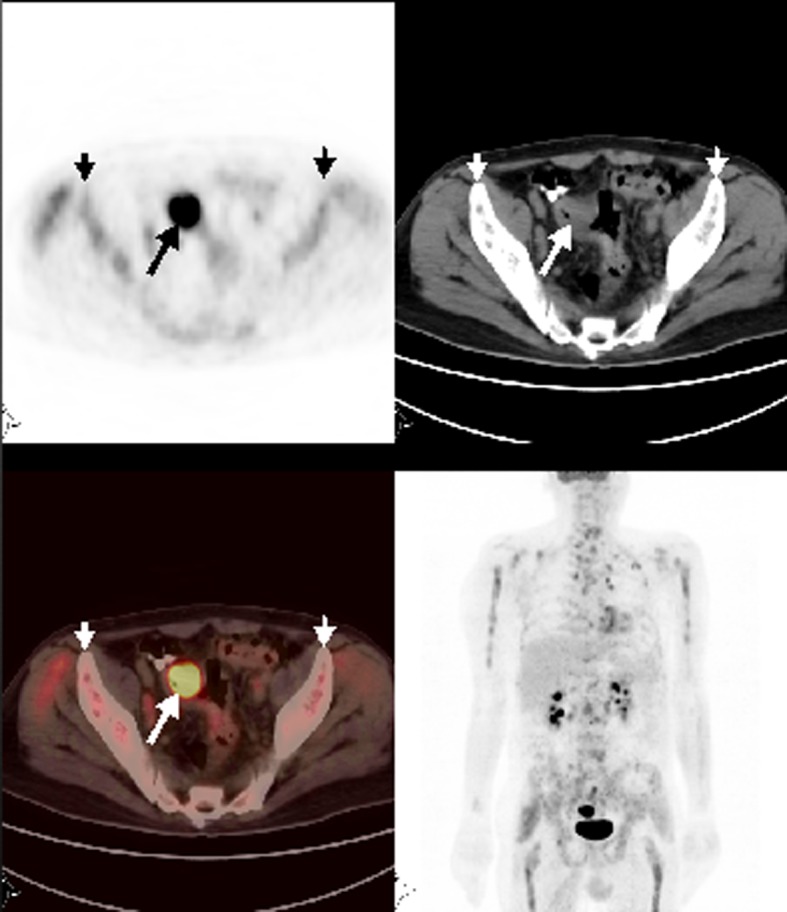
Axial and MIP PET/CT showed a mass with intense FDG uptake at the sigmoid colon consistent with primary tumor. Also, there was heterogeneously increased FDG accumulation at the skeleton suggestive for bone marrow metastases.

A 67-year-old man with sigmoid colon cancer was referred for PET/CT for the evaluation of increased tumor marker after one year of diagnosis. FDG-PET/CT revealed tumoral wall thickening with intense FDG uptake at the sigmoid colon consistent with recurrence and metastatic mesenteric and paraaortocaval lymph nodes. In addition, there was widespread heterogeneously increased FDG uptake in the skeleton consistent with bone marrow involvement (Fig. 2). Bone marrow biopsy proved mucinous tumor involvement despite a platelet count of 179,000 per µL.

### Patient 5

A 68-year-old male presented with right shoulder pain for the last 4 weeks. Thorax CT revealed a mass at the level of right upper lobar bronchus in the lung. Bronchoscopic biopsy showed small cell lung cancer. FDG-PET/CT was ordered in order to stage the tumor. PET/CT images demonstrated increased FDG metabolism at the primary tumor with multiple metastatic lymphadenopathies and liver metastases. Additionally, there was widespread heterogenous FDG uptake in the axial skeleton reminiscent of bone marrow metastases. Complete blood count was consistent with mild thrombocytopenia (101,000 per microliter). A trephine biopsy from the posterior iliac crest done when the thrombocyte count dropped to 49,000 after one week proved that there was bone marrow involvement with small cell lung cancer. This case beautifully illustrates that PET/CT heralded bone marrow involvement before we suspected from complete blood count.

### Patient 6

A 44-year-old male surgeon was presented to the neurology clinic with headache. After searching for possible causes of headache, pseudotumor cerebri was diagnosed. Mild anemia, decreased albumin, increased alkaline phosphatase and lactate dehydrogenase prompted to search for malignancy. Low platelet value together with anemia, decreased fibrinogen and increased D-dimer levels were consistent with microangiopathic hemolytic anemia and disseminated intravascular coagulation. Abdominal and pelvic CT revealed left adrenal mass, gastrohepatic lymphadenomegaly and a left iliac lytic lesion. FDG-PET/CT which was ordered for detection of primary site revealed moderately hypermetabolic lesion at the left upper abdomen suggestive of primary gastric tumor, in addition to widespread axial bone and bone marrow metastases, celiac, paraaortocaval and mesenteric lymphadenopaties with increased FDG enhancements. Upper endoscopic study proved gastric signet-ring cell carcinoma. A bone marrow trephine biopsy showed infiltration of the marrow with signet-ring cancer cells. He passed away without having chance for a chemotherapy challenge due to high level of bilirubinemia.

### Patient 7

**Figure 3:**
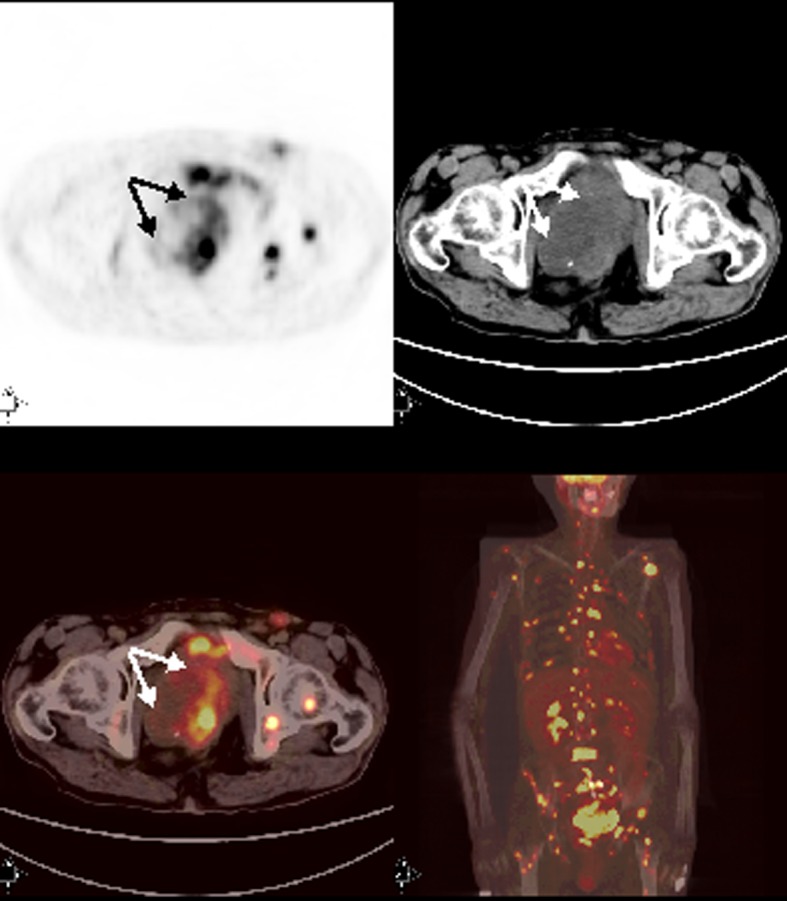
Axial and MIP PET/CT images showed primary tumor with intense FDG accumulation in rectosigmoid. There were also multipl bone-bone marrow metastases and extraskeletal metastases with prominent FDG uptake.

A 56-year-old male patient who was on follow-up for rectosigmoid colon cancer revelaed a pelvic mass on abdominal CT. FDG-PET/CT performed for staging showed hypermetabolic mass consistent with recurrence and metastatic lymphadenopaties at the left inguinal, right external iliac and promontorial region. PET/CT also revealed metastatic lesion in the liver and multipl lesions at the bones as well as in the bone marrow (Fig. 3). Bone marrow biopsy was done with absolutely normal blood count directed by PET/CT findings. Bone marrow metastasis was found consistent with poorly differentiated mucinous adenocarcinoma secondary to colon cancer.

### Patient 8

**Figure 4:**
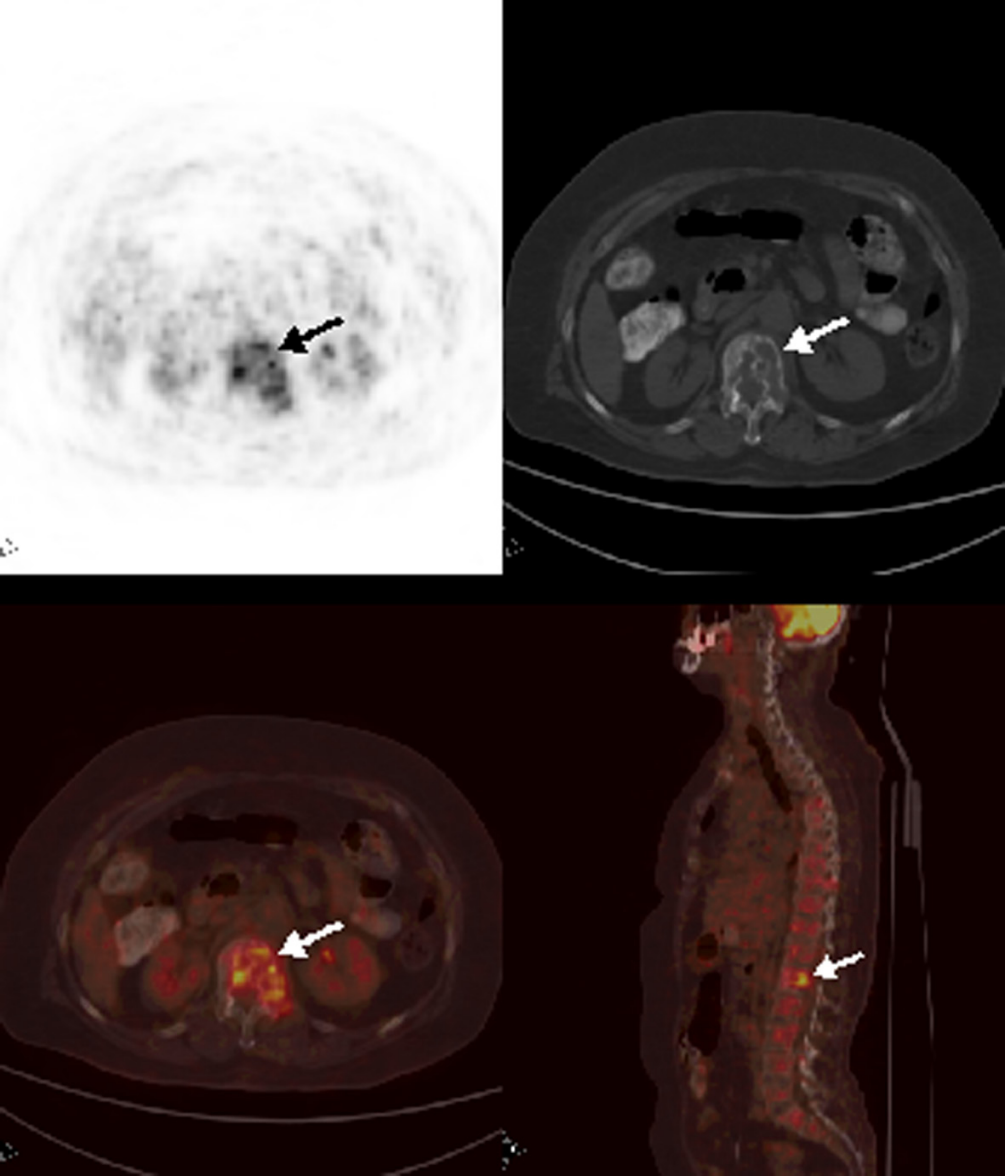
Axial and sagittal PET/CT images depicted multiple heterogeneously increased FDG uptake at vertebral column.

A 69-year-old woman with nasopharyngeal cancer was referred to PET/CT in order to detect a suspected local recurrence. FDG PET/CT images demonstrated increased FDG uptake at the left nasopharyngeal region suggestive of locally recurrent tumor (Fig. 4). Also, there were multiple bone and bone marrow involvement at the vertebral column and manubrium sterni. Bone marrow biopsy proved sinonasal tumor involvement despite a thrombocyte count of 180,000 per µL.

### Patient 9

In a 52-year-old man with gastric signet-ring cell tumor FDG PET/CT was performed for initial staging purposes. PET/CT study showed slightly increased FDG uptake at the gastric wall as well as diffuse lytic lesions with slightly heterogeneous increased FDG uptake suggestive of metastases. Despite normal complete blood count indices bone marrow biopsy revealed signet-ring cell carcinoma infiltration.

### Patient 10

**Figure 5:**
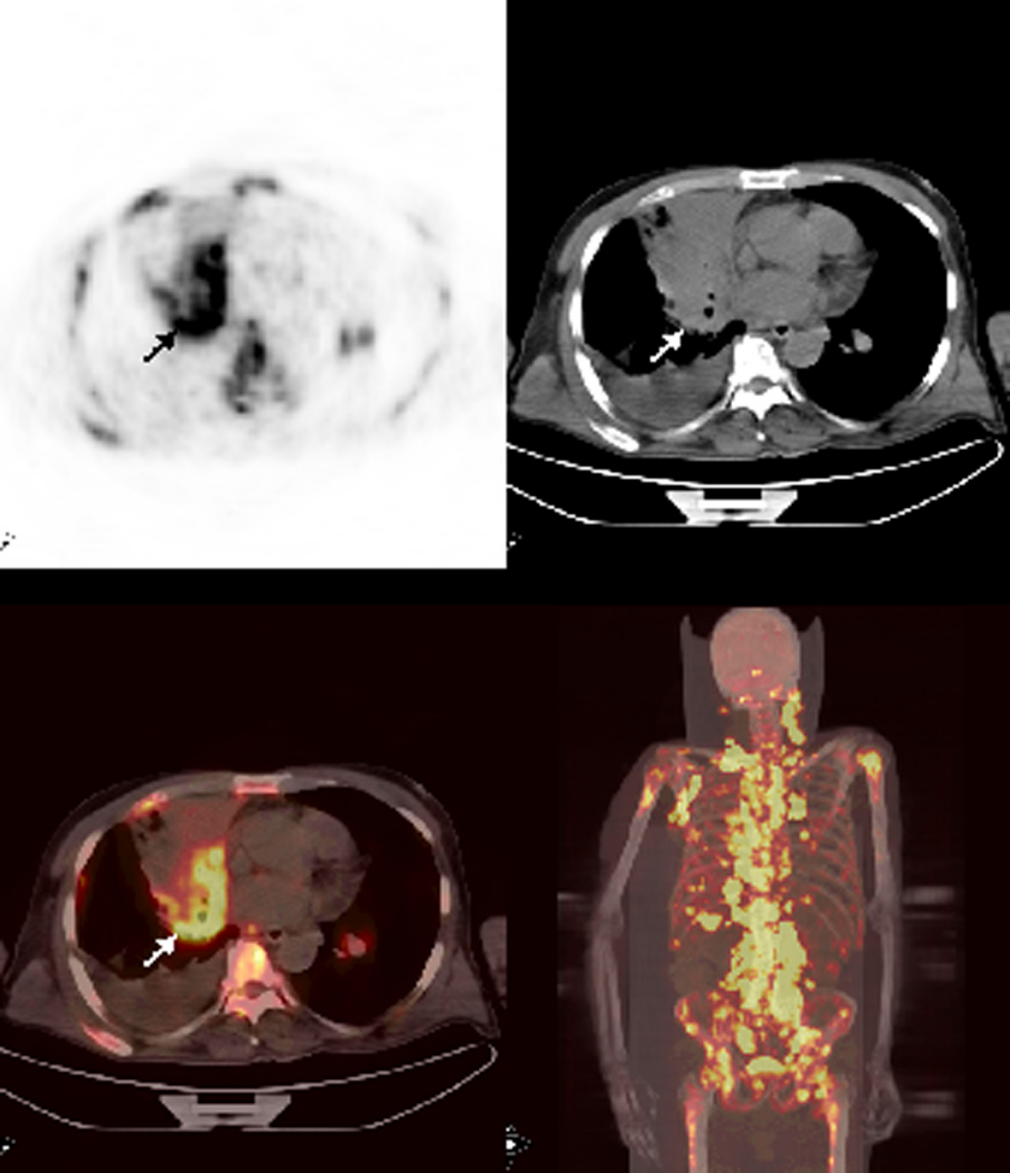
Axial and MIP PET/CT images showed a mass at the right hilar region with very intense FDG accumulation. There were also multipl bone-bone marrow metastases and extraskeletal metastases with prominent FDG uptake.

A 62-year-old male patient with history of chronic lymphocytic leukemia presented with cough, shortness of breath, cervical lymphadenopathy and sweating for the last 3 months. A cervical excisional biopsy showed undifferentiated carcinoma and infiltration with small lymphocytes consistent with chronic lymphocytic leukemia. A PET/CT which was ordered for staging and detection of primary tumor revealed hypermetabolic mass at the right hilar region. Also, metastatic bilateral cervical, supraclavicular, bilateral axillary, abdominal, pelvic lymphadenopathies in addition to multiple liver and widespread bone marrow metastasis were detected (Fig. 5). Although complete blood count was normal, diffuse bone marrow involvement prompted us to attempt a bone marrow biopsy. Biopsy showed poorly differentiated large cell carcinoma of the lung.

## Results

FDG-PET/CT was ordered in 10 out of 68 solid tumor patients with pathologically proven BMM. Of these 10 patients, 3 were female and 7 were male; mean age was 54.7 years (range 24 - 69). While FDG PET/CT showed bone and BMM in 4 of 10 patients (40%), the rest of the patients had BMM without bone involvement. In other words BMMs were detected in all of our patients. There was a wide variation of tumor types. The characteristics of the patients are presented in Table 1. Five patients (50%) who had probable bone marrow involvement on their FDG PET/CT scans had unsuspected complete blood counts with regard to BMM. Bone marrow biopsies showed 5 adenocarcinoma, 4 of which were mucinous in type, 1 sinonasal cancer, 1 rhabdomyosarcoma, 1 small cell lung cancer, 1 large cell lung cancer and 1 breast carcinoma.

**Table 1:** Patient Characteristics

Patient No.	Sex	Age	Diagnosis	LV/L	LV/Spleen	IW/KC	IW/Spleen	SB/L	SB/Spleen	Bone Met	Hemogram		
											WBC	HGB	PLT
1	male	46	Gastric ca	3.17	1.9	2.6	1.6	2.31	1.42	No	4.1	9.5	33
2	female	24	Rhabdomyosarcoma	2.9	3.7	2.86	3.48	2.75	3.35	Yes	7.2	10.5	92
3	female	59	Breast ca	1.14	1.4	1.44	1.77	1.79	2.2	No	7.4	9.6	78
4	male	67	Colon ca	2.1	2.16	1.69	1.75	2.21	2.28	No	5.3	11	179
5	male	68	NSCLC*	3.14	4	3.81	4.86	23	2.93	No	5.6	11.6	49
6	male	44	Gastric ca	2.05	2.3	2.03	2.27	1.92	2.15	Yes	13	9	26
7	male	56	Colon ca	4	5.17	3	3.91	3.96	5.17	Yes	11	14	314
8	female	69	Nasopharyngeal ca	2.6	2.83	1.72	1.9	1.94	2.14	Yes	4.5	12	180
9	male	52	CUP**	1.16	1.69	1.08	1.58	0.71	1.04	No	16	13.4	207
10	male	62	NSCLC, CLL	4.5	7.26	4.46	7.13	5.96	9.53	No	39	10.4	130

*Non-small cell lung cancer; **Carcinoma of unknown primary; LV: Lomber Vertebra; L: Liver; IW: Iliac Wing; SB: Sternal Body.

## Discussion

Bone metastases are a frequent complication of cancer, occurring in up to 70 percent of patients with advanced breast or prostate cancer and in approximately 15 to 30 percent of patients with carcinoma of the lung, colon, stomach, bladder, uterus, rectum, thyroid, or kidney [7]. By applying sensitive immunocytochemical and molecular assays, disseminated tumor cells (DTC) in bone marrow (BM) can be detected in 20-40% of cancer patients without any clinical or even histopathological signs of metastasis [8]. Detection of overt bone marrow metastasis in adult patients with solid tumors might change the approach to the patient especially without any symptoms and signs of marrow infiltration as there is evidence, at least from patients with breast [9] and gastric cancer [10], even micrometastatic disease in bone marrow is associated with a poor prognosis.

FDG-PET has emerged as a powerful modality for evaluating skeletal metastasis and revolutionized the diagnosis and staging of patients with solid tumors especially when other diagnostic imaging methods such as bone scintigraphy, CT or MRI results are equivocal. Because tumor cells have increased rate of glucose metabolism metastatic lesions can be detected by FDG-PET/CT earlier than the time of altered anatomy.

Although bone scintigraphy has traditionally been accepted as the diagnostic modality of choice to assess bone metastasis, it has the disadvantage of missing early bone marrow metastasis due to absence or minimal reactive bone formation. For example in one recent study, 20% of patients with NSCLC had absolutely normal Tc-99m bone scintigraphy while PET/CT showed involvement. In addition, lytic lesions are not detected by bone scintigraphy due to the absence of osteoblastic reaction in some solid tumors [11]. Attempts to overcome this problem have resulted in the utilization of bone marrow scintigraphy in addition to bone scanning has not also been gained popularity because of relatively high false positive [12] and false negative [13] results. CT has also a similar drawback with bone scintigraphy in delineating bone marrow metastases because of the absence of reactive bone formation or cortical destruction in lesions confined to bone marrow. MRI has recently been regarded as a very sensitive modality in detecting BM metastasis compared to bone scintigraphy due to fact that it has the ability to show malignant deposits directly before reactive changes have occurred [14]. Daldrup-Link et al demonstrated that whole-body MRI, FDG PET and skeletal scintigraphy has a sensitivity of 82%, 90% and 71%, respectively in detecting skeletal metastases [15]. On the other hand, MRI has been considered to be not specific enough in delineating bone marrow metastases due to some benign entities such as hemangiomas, inflammatory processes, and post-therapy changes and also impractical for various reasons such as absence of widespread availability or claustrophobia of the patients [16, 17]. The superiority of FDG-PET/CT lies in the fact that it has the ability to evaluate not only the entire skeleton but also the other organs in a single examination. Many studies have confirmed that FDG-PET/CT has comparable sensitivities in the ability of detecting bone marrow metastases in solid tumors [18, 19]. It has also shown efficacy for the detection of BM metastases from lung and breast carcinoma [20, 21]. The diagnostic accuracy, sensitivity and specificity of FDG-PET in detecting bone marrow metastasis in lung cancer were 94%, 91% and 96%, respectively, compared to bone scintigraphy in one study [20]. FDG-PET is also superior to other modalities in identifying unsuspected disease involving the bone at an earlier stage when only the bone marrow has been involved before bone remodeling has occurred. This might pose many clinical implications. Some solid tumors such as prostate and breast have predilection to metastasize to bone relatively early to their course of disease. Early detection of bone and BM metastasis may change the treatment plan dramatically both for bone directed therapies such as zoledronic acid or denosumab and radiotherapy and for systemic therapies. For example, detecting bone and BM metastasis in otherwise early stage hormone positive breast cancer may obviate the need for combination chemotherapy or establishing osteoclast inhibiting (zoledronic acid, ibandronic acid) or RANK ligand inhibiting (donesumab) therapies earlier than overt metastases. Instead, we can put the patient on hormonotherapy which is far less toxic than chemotherapy. In our patients, after ordering PET/CT for other reasons bone and bone marrow metastases were detected unexpectedly, despite normal complete blood count indices in 5 patients (patient 4, 7, 8, 9, and 10). This prompted us to modify the management plan as follows: In patient 4, 7 and 10, finding of bone marrow metastasis led to a decrease in the initial chemotherapy doses to avoid severe bone marrow toxicity. In patient 8 and 9, detecting bone and bone marrow metastasis obviated the need of local therapy. Instead, we planned bisphosphonate and tailored chemotherapy for this patient.

On the other hand, in those patients with abnormal blood counts proving bone metastasis, supported by PET/CT scans, allowed us to administer chemotherapy, if indicated, without jeopardizing our medicolegal status (patient 1, 2, 3, 5 and 6).

At sites where there is increased FDG accumulation relative to other sites the diagnostic yield of BM biopsies can increase dramatically at least theoretically. Many studies such as that of Moog’s et al point out that FDG-PET facilitates detecting accurate biopsy sites in lymphomas which have metastasized to the bone marrow [4]. However, standardized biopsies from the posterior iliac crest were done in all of our patients.

Despite the advantages of FDG PET/CT, it has some drawbacks that need to be born in mind. Granulocyte colony stimulating factor (G-CSF) for BM stimulation may result in diffusely increased FDG accumulation in the BM which can cause misinterpretation as generalized marrow metastases. In addition, small metastatic lesion in the marrow can be missed due to benign bone marrow hyperplasia (such as G-CSF or presence of anemia) or inherent resolution limits of PET/CT. We found that all of our cases had increased FDG uptake in their bone marrows suggesting bone marrow involvement. FDG-PET/CT, furthermore, has a lower sensitivity in detecting sclerotic metastases seen particularly in prostate cancer probably because of hypocellularity although we did not have any case of prostate cancer to exemplify this fact [2]. Osteoblastic metastases of breast cancer have a low or undetectable FDG uptake [22].

### Conclusion

Finding of bone marrow metastases might have many clinical implications as we depicted in our patients summaries. PET/CT has the ability to detect a substantial number of metabolically active tumor cells in the bone marrow in all of our patients which we proved by bone marrow biopsies. We think that this cohort of patients with solid tumors is hypothesis-generating with regard to detecting early bone marrow metastases by FDG PET/CT.
